# Predictors and correlations of emotional intelligence among medical students at King Abdulaziz University, Jeddah

**DOI:** 10.12669/pjms.335.13157

**Published:** 2017

**Authors:** Nahla Khamis Ibrahim, Wafaa Ali Algethmi, Safia Mohammad Binshihon, Rawan Aesh Almahyawi, Razan Faisal Alahmadi, Maha Yousef Baabdullah

**Affiliations:** 1 Nahla Ibrahim, Professor of Epidemiology at Family & Community Medicine Department, Faculty of Medicine, King Abdulaziz University, Jeddah, Saudi Arabia. Professor at Epidemiology Department at High Institute of Public Health, Alexandria University, Alexandria, Egypt; 2 Wafaa Algethmi, Sixth Year Medical Student, King Abdulaziz University, Jeddah, Saudi Arabia; 3 Safia Binshihon, Sixth Year Medical Student, King Abdulaziz University, Jeddah, Saudi Arabia; 4 Rawan Almahyawi, Sixth Year Medical Student, King Abdulaziz University, Jeddah, Saudi Arabia; 5 Razan Alahmadi, Sixth Year Medical Student, King Abdulaziz University, Jeddah, Saudi Arabia; 6 Maha Baabdullah, Sixth Year Medical Student, King Abdulaziz University, Jeddah, Saudi Arabia

**Keywords:** Emotional intelligence, Predictors, Leadership, Self-efficacy, Perceived-stress

## Abstract

**Objectives:**

To determine the predictors of Emotional Intelligence (EI), and its relationship with academic performance, leadership capacity, self-efficacy and the perceived stress between medical students at King Abdulaziz University, Jeddah, Saudi Arabia.

**Methods:**

A cross-sectional study was done among 540 students selected through a multi-stage stratified random sampling method during 2015/2016. A standardized, confidential data collection sheet was used. It included Schutte Self-Report Emotional Intelligence (SSREI) scale, Authentic Leadership questionnaire, General Self-Efficacy Scale and the short version of Perceived Stress Scale (PSS-4). Both descriptive and inferential statistics were done, and a multiple linear regression model was constructed.

**Results:**

The predictors of high EI were gender (female), increasing age, and being non-smoker. EI was positively associated with better academic performance, leadership capacity and self-efficacy. It was negatively correlated to perceived-stress.

**Conclusion:**

Female gender, age, non-smoking were the predictors of high EI. Conduction of holistic training programs on EI, leadership and self-efficacy are recommended. More smoking control programs and stress management courses are required.

## Introduction

Emotional intelligence (EI) plays an important role in medicine and medical education.^1^-^3^ EI is defined as the ability to perceive emotions, access and generate emotions, understand emotions, and reflectively regulate it for promoting emotional and intellectual growth.^2^ It denotes the cooperative mixture between intelligence and emotions. It can contribute to individual cognitive-based performance equal or may be better than Intelligence Quotient (IQ).^4^,^5^

EI has been recognized as an essential trait for medical students and health care providers.^1^,^6^ It can be considered as one predictor of good performance and better quality of care delivered to patients. Medical students, as future practitioners, need to have sound EI for having more effective communication and empathy with their patients.^2^,^7^

Self-efficacy is one of the social cognitive function and defined as one’s belief in one’s ability to succeed at tasks. Self-efficacy is suggested to be related to EI. Furthermore, self-awareness, self-regulation, motivation, empathy, and social skills are necessary components of effective leadership. EI can improve the features necessary for a worthy and influential leader.^8^ However, medical students are exposed to many stresses, especially the academic stress, which may affect EI.^6^

Emotions are essential determinants of how well medical students function. However, EI is seen as a long-neglected core component of mental ability.^9^ There is lack of such epidemiological studies among medical students in Jeddah.

This study was done to determine the predictors of EI, and its relationship with academic performance, leadership capacity, self-efficacy and the perceived stress between medical students at King Abdulaziz (KAU) University, Jeddah, Saudi Arabia.

## methods

A cross-sectional study was conducted at King Abdulaziz University (KAU), Jeddah, during the educational year 2015/2016. Medical students who completed the freshman year (2^nd^ - 6^th^ year) and accepted to participate were recruited. A multistage stratified random sample method was used (stratification was done by gender and educational year). The sample size was calculated according to formula:^10^


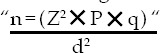


We assumed that p = 0.5 (as the most conservative sample). So, “q” = 1–p = 1–0.5 = 0.5, d was put as 0.042. The minimal calculated sample was 544 (rounded to 540). A confidential, anonymous, self-administered data collection sheet was used and asked about:

1. **Personal and socio-demographic data**
2. **Habits as** smoking and practicing exercise.
3. **The English version of Schutte Self-Report Emotional Intelligence** (SSREI). Participants rated their agreement on 33 questions through 5-point Likert scale.^11^,^12^ SSREI scale has good internal consistency & test-retest reliability.^11^ SSREI composed of 4 sub-scales which are: Emotion Perception (EP), Managing Self-Relevant Emotions (MSE), Managing Others’ Emotions (MOE) and Utilizing Emotion (UE).^11^,^12^
4. **Authentic Leadership Self-Assessment Questionnaire**: It contains 16 items and measures 4 subscales (self-awareness, internalized moral, balanced processing and relational transparency).^13^
5. **General Self-Efficacy Scale:** It consists of 10 items with response on 4-point Likert scale. The Cronbach’s α was range from 76% - 90%.^14^
6. **The short version of Perceived Stress Scale (PSS-4)**: It contains 4 items based on 5-point Likert-type scale.^15^

The face and content validity of the sheet was assessed by 2 experts. Internal-consistency reliability was assessed with Cronbach’s alpha and was found to be 81%. The calculated Cronbach’s alpha was 91% for SSREI, 81.2% for Authentic Leadership Self-Assessment Questionnaire, 84% for General Self-Efficacy Scale was and 65% for PSS-4.

Statistical analysis was done using SPSS version 21. The total EI score, and each of its four sub-scales were calculated.^11^,^12^ Furthermore, Authentic Leadership Self-Assessment score (with 4 sub-scales)^13^, General Self-Efficacy Scale^14^ and PSS-4^15^ were computed. Descriptive statistics was performed. Student’s t-test, One-way ANOVA with Least Significant Differences (LSDs), and Pearson’s Correlations were done. A multiple linear regression model was constructed to determine the predictors of EI. *P*-values < 0.05 were considered statistically significant.

### Ethical Statement

The study was conformed to ethical standards of “Helsinki Declaration”. It was approved by the Institutional Review Board (IRB) of KAU, with Reference Number: 34-16. A written consent was taken from participants. Administrative approvals were taken, and approval for using SSREI scale was obtained from the author.

## Results

A total of 540 students participated in the study. Their mean age was 21.42 ± 1.48 years. The total EI mean score was 116.22±16.12.

Females obtained significantly better mean scores of all EI domains compared to males (*p* <0.001), as shown in Table-I. The table also shows that older students, those in higher educational years and the better achievers (those obtained higher GPAs) had significantly better EI scores compared to others. MOE domain of EI was significantly associated with residency (F = 3.07; *p* < 0.05). Students living in private dorms had significantly lower mean EI score than those living with families. Smokers had significantly lower scores in three EI domains (MSE, MOE, and UE) compared to non-smokers. However, those practicing physical activities obtained higher EI score than others. Education and occupation of both parents didn’t affect students’ EI.

**Table-I:** Mean differences between emotional intelligence domains according to study variables, among medical students in King Abdulaziz University

*Variables*	*Emotional intelligence*								*Total EI*	

	*Perception of emotion*		*Managing self-emotions*		*Managing others’ emotions*		*Utilizing emotions*			

	*Mean*	*SD*	*Mean*	*SD*	*Mean*	*SD*	*Mean*	*SD*	*Mean*	*SD*
***Gender:***										
Male (n= 182)	33.53±5.71		29.57±5.91		27.24±5.62		20.65±4.47		111.0 ±18.20	
Female (n = 358)	34.51±4.78		31.81±5.01		29.70±4.66		22.83±4.02		118.88±14.26	
Student’s t test, (p)	-2.00	(0.04)	- 4.38	(0.000)	-5.09	(0.000)	-5.54	(0.000)	- 5.51	(0.000)
***Age:***										
< 21 (n= 305)	33.56±5.00		30.43±5.19		28.17±4.96		21.31±4.22		113.47±15.42	
≥ 21 (n= 235)	35.00±5.19		31.87±5.64		29.79±5.23		23.13±4.19		119.80±16.34	
Student’s t test, (p)	-3.28	(0.001)	-3.08	(0.002)	-3.69	(0.000)	-4.97	(0.000)	- 4.61	(0.000)
***GPA:***										
≤ 4 (n=149)	33.46±5.62		30.29±6.11		28.17±5.30		21.64±4.27		113.56±17.53	
> 4 (n=361)	34.59±4.86		31.47±4.93		29.22±4.80		22.28±4.31		117.57±14.89	
Student’s t test, (p)	-2.30	(0.022)	-2.09	(0.037)	-2.17	(0.03)	-1.54	(0.124)	-2.62	(0.009)
***Nationality:***										
Saudi^a^ (n=501)	34.19±5.13		31.02±5.51		28.91±5.11		22.08±4.30		116.19±16.26	
Yamani^b^ (n= 8)	33.62±3.42		31.00±3.02		27.38±3.11		21.63±2.26		113.63±8.07	
Egyptian^c^ (n= 11)	35.91±5.74		33.36±4.82		32.36±3.98		23.45±4.78		125.09±9.93	
Others^E^ (n=20)	33.15±5.54		30.75±4.44		26.65±6.05		21.90±4.79		112.45±16.55	
ANOVA F test, (p)	0.72 (0.54)		0.69 (0.559)		3.21 (0.02)		0.41 (0.745)		1.55 (0.20)	
**LSD**	^c^statistically differs from others									
***Educational year:***										
Second year (n=136)	32.68±4.76		29.98±4.98		27.62±4.75		20.74±4.27		111.01±14.80	
Third - sixth year (n=404)	34.69±5.15		31.42±5.53		29.30±5.20		22.56±4.21		117.98±17.61	
Student’s t test, (p)	-4.05	(0.000)	-2.69	(0.007)	-3.34	(0.001)	4.35	(0.000)	-4.43	(0.000)
***Smoking:***										
Yes (n= 69)	33.45±5.19		29.38±5.84		26.72±5.59		20.32±4.28		109.87±17.59	
No (n= 471)	34.29±5.12		31.30±5.33		29.19±4.99		22.36±4.25		117.16±15.69	
Student’s t test, (p)	-1.28	(0.20)	-2.77	(0.006)	-3.77	(0.000)	-3.73	(0.000)	-3.54	(0.000)
***Physical activity:***										
Yes (n=287)	34.10±5.19		31.36±5.49		29.03±5.47		22.23±4.42		116.72±17.05	
No (n=253)	34.28±5.07		30.71±5.35		28.71±4.75		21.96±4.17		115.65±15.01	
Student’s t test, (p)	-0.39	(0.69)	1.39	(0.16)	0.72	(0.47)	0.74	(0.46)	0.76	(0.44)
***Place of residence:***										
With family^a^ (n= 480)	34.18±5.18		31.19±5.50		29.03±5.22		22.19±4.28		116.59±16.26	
Univ. dorm^b^ (n= 23)	34.43±5.57		31.48±3.55		29.04±4.01		22.09±3.96		117.04±10.65	
Private dorm^c^ (n= 37)	34.11±5.47		29.14±5.25		26.86±4.37		20.92±4.65		111.03±16.47	
ANOVA F test, (p)	0.03	(0.97)	2.53	(0.08)	3.07 (0.04)		1.51	(0.22)	2.08 (0.13)	
**LSD**					^a^ statistically differs from^c^					
***Total domains***	34.18±5.13		31.06±5.43		28.88±5.43		22.10±4.30		116.22±16.12	

Results of linear regression analysis (Table-II) shows that the first predictor of better EI was the female gender (t= 5.19; *p* < 0.001), followed by increased age, and being non-smoker.

**Table-II:** Multiple linear regression analysis of predictors of total emotional intelligence score among medical students in King Abdulaziz University.

*Predictors*	*B*	*Standardized Beta*	*t-test*	*Sig. “*p*”*	*R^2^*	*F*	*Sig.*
Constant	83.05						
Gender (Female)	7.460	0.219	5.19	0.000			
Age	5.486	0.169	3.60	0.000	0.112	16.79	0.000
Non-smoking	4.07	0.084	1.99	0.04			

Table-III reveals that EI sub-scales had moderate positive correlations with each other. The strongest correlation was found between MSE and MOE (r=0.608). This is followed by UEI and MOE (r=0.599) and UEI with MSE (r=0.573). Table-IV illustrates presence of positive correlations between EI domains with each of authentic leadership and self-efficacy (*p* <0.001). On the other hand, negative correlations were found between different EI domains and the students’ perceived stress.

**Table-III:** Correlations between different emotional intelligence domains among medical students at King Abdulaziz University.

*EI domains*	*Perception of emotion*	*Managing self-emotion*	*Managing others’ emotion*	*Utilizing emotion*
Perception of emotion	1	0.454**	0.512**	0.444**
Managing self-emotion		1	0.608**	0.573**
Managing others’ emotion			1	0.599**
Utilizing emotion				1

** means *p* < 0.01

**Table-IV:** Correlations between different emotional intelligence domains with leadership, self-efficacy and perceived-stress scales

	*Emotional intelligence domains*			

*Scales*	*Perception of emotion*	*Managing self-emotion*	*Managing others’ emotion*	*Utilizing of emotion*
Leadership (r)	0.516**	0.510**	0.571**	0.527**
Self-efficacy (r)	0.426**	0.553**	0.443**	0.462**
Perceived- stress (r)	-0.111*	-.286**	-.138**	-0.165**

* means p < 0.05 and

** means p < 0.01.

Presence of positive correlations between all EI domains and authentic leadership subscales are shown in Table-V. The highest association was found between UE domain of EI and self –awareness leadership scale (r= 0.5, *p* < 0.05).

**Table-V:** Correlations between emotional intelligence domains and leadership sub-scales among medical students at King Abdulaziz University.

	*Emotional intelligence domains*			

*Leadership sub-scales*	*Perception of emotion*	*Managing self-emotion*	*Managing others’ emotion*	*Utilizing of emotion*
Self-awareness (r)	0.467**	0.475**	0.487**	0.500**
Internalized moral (r)	0.431**	0.426**	0.436**	0.411**
Balanced processing (r)	0.380**	0.363**	0.435**	0.413**
Relational transparency (r)	0.361**	0.356**	0.459**	0.353**

** means: *p* < 0.01

## Discussion

Our results revealed that gender was the first predictor of high EI; females obtained significantly better EI scores compared to males. This result coincides with many other studies.^3^,^16^,^17^ This finding may be attributed to more socialization, societal expectations, girls’ motherly nature, and better learning emotions by females (as parents usually share emotional conversation and utilize extra-emotional terms with their daughters more than sons).^18^ On the other hand, results of studies done among university students from Albaha, Saudi Arabia,^19^ and between Medical Sciences students from Iran^20^ revealed absence of such association. These dissimilarities may be due to differences between sample sizes, target populations, or other socio-cultural factors. In some societies females may have limited access to social environments and other public societies compared to males.^20^

In the current study, EI scores improved with the increasing students’ age and educational year, which agrees with results from India.^21^ This may be because EI skills can be learned, and strengthened by increasing life experience.^22^

Our results also showed presence of positive association between EI and better academic performance, which coincides with many other studies.^3^,^7^,^20^,^23^,^24^ EI can be seen as an ability-based skill that allows training in specific competencies which lead to fewer learning problems and better academic achievement.^2^ On the other hand, Albaha study reported absence of such association.^19^ This discrepancy may be attributed to their sample size.

Being non-smoker was a predictor of better EI. Persons with high EI seems to have more constructive conflict resolution strategies,^25^ so they are usually non-smokers. Our findings also revealed that MOE domain of EI was better among students living with their families than those living in private dorms. This may be due to better communication between family members.

In our study, positive correlations were found between different EI facets together, which agree with results of Brackett, et al.^26^ Similarly, we found positive correlation between EI score and self-efficacy, which agrees with another Iranian study.^9^

Our results illustrated that EI domains was positively associated with leadership capacity, which coincides with other studies.^27^,^28^ Furthermore, many previous researches^3^,^21^,^29^ have demonstrated negative association between EI and the perceived stress, which agrees with our findings. Hence, effective regulation of emotional response is important for mitigating impact of stress^17^, and stress management can improve EI.

### Strengths and clinical implications of the study

Up to the best of our knowledge and based on extensive literature search, our study may be the first study done to determine the predictors and correlations of EI among a large sample of medical students in Jeddah. Regarding clinical implication, EI was associated with better students’ performance, leadership capacity and self- efficacy. So, implementation of EI training within the medical school curricula is expected to improve future physicians’ overall capacities (as interpersonal and communication skills), while caring for their patients. This can enhance patients’ experience and improve patients’ care and outcomes.

### Limitation of the study

**PSS-4** had a moderate reliability.

## CONCLUSION

Female gender, increasing age and being non-smoker were EI predictors. EI sub-scales had positive correlations with each other. EI was positively associated with better academic performance, leadership capacity and self-efficacy. It was negatively correlated to perceived stress. Training on EI is recommended to be an integral part of medical curricula. This training needs to be interdisciplinary and as a holistic part of the existing curricula. This can be done through practical and applied preparation. Furthermore, students need to have opportunities to practice EI skills. EI intervention studies need to be done through the involvement of EI within medical curriculum. In addition, more training is required for improving students’ leadership capacity and self-efficacy through curriculum and extra-curriculum activities. Conduction of stress management courses and intensifying smoking control programs are required.
